# Quantifying the Importance of MSP1-19 as a Target of Growth-Inhibitory and Protective Antibodies against *Plasmodium falciparum* in Humans

**DOI:** 10.1371/journal.pone.0027705

**Published:** 2011-11-15

**Authors:** Danny W. Wilson, Freya J. I. Fowkes, Paul R. Gilson, Salenna R. Elliott, Livingstone Tavul, Pascal Michon, Elija Dabod, Peter M. Siba, Ivo Mueller, Brendan S. Crabb, James G. Beeson

**Affiliations:** 1 The Walter and Eliza Hall Institute of Medical Research, Parkville, Australia; 2 University of Melbourne, Melbourne, Australia; 3 Macfarlane Burnet Institute for Medical Research and Public Health, Melbourne, Australia; 4 Monash University, Clayton, Australia; 5 Papua New Guinea Institute of Medical Research (PNGIMR), Goroka, Papua New Guinea; 6 Faculty of Health Sciences, Divine Word University, Madang, Papua New Guinea; 7 Barcelona Centre for International Health Research (CRESIB), Barcelona, Spain; Université Pierre et Marie Curie, France

## Abstract

**Background:**

Antibodies targeting blood stage antigens are important in protection against malaria, but the key targets and mechanisms of immunity are not well understood. Merozoite surface protein 1 (MSP1) is an abundant and essential protein. The C-terminal 19 kDa region (MSP1-19) is regarded as a promising vaccine candidate and may also be an important target of immunity.

**Methodology/Findings:**

Growth inhibitory antibodies against asexual-stage parasites and IgG to recombinant MSP1-19 were measured in plasma samples from a longitudinal cohort of 206 children in Papua New Guinea. Differential inhibition by samples of mutant *P. falciparum* lines that expressed either the *P. falciparum* or *P. chabaudi* form of MSP1-19 were used to quantify MSP1-19 specific growth-inhibitory antibodies. The great majority of children had detectable IgG to MSP1-19, and high levels of IgG were significantly associated with a reduced risk of symptomatic *P. falciparum* malaria during the 6-month follow-up period. However, there was little evidence of PfMSP1-19 specific growth inhibition by plasma samples from children. Similar results were found when testing non-dialysed or dialysed plasma, or purified antibodies, or when measuring growth inhibition in flow cytometry or microscopy-based assays. Rabbit antisera generated by immunization with recombinant MSP1-19 demonstrated strong MSP1-19 specific growth-inhibitory activity, which appeared to be due to much higher antibody levels than human samples; antibody avidity was similar between rabbit antisera and human plasma.

**Conclusions/Significance:**

These data suggest that MSP1-19 is not a major target of growth inhibitory antibodies and that the protective effects of antibodies to MSP1-19 are not due to growth inhibitory activity, but may instead be mediated by other mechanisms. Alternatively, antibodies to MSP1-19 may act as a marker of protective immunity.

## Introduction

Between 300–500 million cases of clinical malaria occur each year resulting in approximately 1 million deaths, mostly in children under 5 years of age in sub Saharan Africa [1]–[3]. The major causative agent of malaria mortality is *Plasmodium falciparum*. Although the disease burden of *P. falciparum* is considered to fall mainly in Africa, around 25% is thought to occur in Asia [4].

Malaria disease occurs during blood-stage infection when merozoites invade human red blood cells (RBCs) and replicate inside them. Invasion of RBCs is a complex process involving the interaction of proteins present on the surface and within apical organelles of the merozoite with receptors on the RBC surface (reviewed in Gaur *et al.* 2004 [5]). In malaria-endemic areas effective immunity against malaria develops after repeated exposure that limits blood-stage parasitemia and prevents symptomatic illness and severe complications [6]. Antibodies to merozoite proteins appear to be an important component of acquired immunity in humans and high levels of IgG to a number of surface proteins and invasion ligands have been associated with protection from malaria [7]–[22]. Antibodies to merozoite proteins are thought to act by direct inhibition of invasion, and through antibody-dependent cell-mediated immune mechanisms [23], [24]. However, antibody effector mechanisms and the specific targets of functional antibodies are poorly understood.

Merozoite surface protein 1 (MSP1), is a ∼200 kDa GPI anchored merozoite surface protein consisting of 17 blocks of conserved and variable regions [25], [26]. MSP1 is abundant on the merozoite surface and efforts to knock-out the protein have been unsuccessful, suggesting that MSP1 is essential for parasite invasion and/or growth [27], [28]. MSP1 is proteolytically processed into 83-, 30-, 38-kDa, and C-terminal 42-kDa (MSP1-42) fragments just before egress from the schizont. These are shed, leaving only the C-terminal fragment that runs at 19 kDa on a non-reducing gel (termed MSP1-19) on the parasite membrane post invasion [29]–[35]. The function of MSP1 remains unclear, but MSP1-19 has been reported to bind the RBC protein Band 3 [36], and recent studies suggest MSP1-42 interacts with heparin-like molecules on the RBC [37].

A number of studies have shown that high levels of MSP1-19 IgG antibodies measured by ELISA are associated with protection from *P. falciparum* malaria [20], [38]–[43]. Affinity purified MSP1-19 specific human antibodies to whole MSP1-19 and domain II [44] (10–100 fold higher than initial serum concentration), but not domain I [45], of MSP1-19 were reported to inhibit merozoite invasion *in vitro*. Vaccination using recombinant MSP1-19 antigens and passive transfer of MSP1-19 antibodies has been reported to confer protection from malaria disease in both mouse and monkey models of malaria [46]–[50].

Several mechanisms by which MSP1-19 antibodies may mediate protection from malaria have been suggested by vaccine studies. These include antibody binding to the 19 kDa fragment and inhibiting protein function and merozoite invasion of erythrocytes, disruption of secondary processing of the MSP1 precursor, agglutination of merozoites and opsonising parasites for destruction by phagocytic cells [31], [34], [51]–[53].

Efforts to study the role and function of naturally acquired PfMSP1-19 antibodies in protection from *P. falciparum* disease were facilitated by the development of MSP1-19 transgenic parasites. The 19 kDa fragment of *P. falciparum* isolate D10 (MAD-20 allele) was replaced with the 19 kDa region of the rodent malaria *P. chabaudi* (PcMEGF) [54] or with the endogenous D10 MSP1-19 sequence (PfM3′) [27] to act as a wild-type control. Growth of the PcMEGF and PfM3′ lines was found to be inhibited by polyclonal antibodies raised against the recombinant antigens, with no evidence of significant cross inhibitory activity between antigens. This approach provided tools to measure growth inhibitory antibodies *in vitro* that are specific to MSP1-19 [54].

Several studies using these transgenic parasites have reported that PfMSP1-19 specific antibodies are a large component of the total *in vitro* growth inhibitory activity of untreated serum and plasma from malaria-exposed individuals [54]–[60]. In one study people with high levels of PfMSP1-19 specific inhibitory activity in their plasma were less likely to be reinfected with *P. falciparum* after drug treatment [55]. However, other studies using the PcMEGF, or a similar transfectant using *P. yoelii* MSP1-19 (PyMEGF), showed no association between PfMSP1-19 specific growth inhibitory activity and infection status [58], [59]. Interestingly, several studies found that the level of PfMSP1-19 specific growth inhibition correlated poorly with levels of PfMSP1-19 antibodies measured by ELISA [54]–[56], [59], [61]. Importantly, no study has yet examined the association between MSP1-19 inhibitory antibodies and prospective risk of symptomatic malaria.

In this study, functional antibodies to MSP1-19 and the importance of MSP1-19 as a target of acquired immunity were evaluated. The contribution of MSP1-19 specific antibodies to the total invasion-inhibitory activity of acquired antibodies was quantified, and associations between antibodies to MSP1-19 and protection from parasitization, high grade parasitemia, and malarial illness were examined in a pediatric treatment-reinfection study in Papua New Guinea (PNG). Furthermore, differences in functional activity were evaluated among acquired human antibodies and antibodies generated in experimental animals by vaccination with recombinant protein.

## Materials and Methods

### Study participants and design

Samples were collected during a pediatric treatment-reinfection study undertaken from June to December 2004 at the Mugil Elementary School (n = 152), Megiar Elementary School (n = 44) and Megiar Primary School (n = 10), 50 km North of Madang, Madang Province, Papua New Guinea [62]. The study was approved by the Medical Research Advisory Committee (MRAC), Papua New Guinea Ministry of Health, The Walter and Eliza Hall Institute Human Research Ethics Committee and the institutional review board of the Veteran's Affairs Medical Center (Cleveland, Ohio). Written consent was obtained from parents/guardians of all participants. A total of 206 children aged 5 to 14 were enrolled (median 9.3 years; interquartile range 8.1 to 10.3 years).

Enrolment with peripheral blood and data collection was conducted over a two-week period in June 2004 by staff from PNGIMR [62]. After physical examination and baseline bleed, the children were treated with a 7 day course of artesunate, according to national guidelines, to clear any current malarial infections. Over the next 6 months, malaria and parasitemic episodes were identified with fortnightly follow-ups (active surveillance) and review by a trained health worker of any child enrolled in the study who presented with symptoms at the local Mugil Health Centre (passive surveillance). In cases where symptoms indicated a current or recent fever, thin and thick blood smears were prepared to screen for parasite infection and estimation of parasitemia. Children diagnosed with malaria were treated with sulfadoxine/pyrimethamine and amodiaquine to cover *P. falciparum* and *P. vivax* according to national guidelines.

Data relating to time to reinfection after treatment, infecting malaria parasite species as indicated by PCR, parasite density as indicated by microscopy, red blood cell polymorphisms by PCR, history of illness as indicated by symptoms and physical examination were available [62], [63]. Some samples were also collected from PNG adults in the same region, and from residents of Melbourne, Australia, to act as non-malaria-exposed controls.

### Measurement of PfMSP1-19 specific antibodies by ELISA

DNA sequences for *P. falciparum* MSP1-19 and *P. chabaudi* MSP1-19 were ligated into a modified pMal-C2x plasmid (New England Biolabs) to produce MSP1-19 fusions proteins with a N-terminal Maltose Binding Protein (MBP) tag. The fusion proteins were expressed in *Escherichia coli* BL21(DE3) Origami (Novagen, Madison, WI, U.S.A.) and purified via nickel-nitrilotriacetic acid chromatography under native conditions (Qiagen, Valencia, CA, U.S.A.) and were refolded as per Dutta *et al.* [64]. Correct folding of the PfMSP1-19 fusion protein was verified by western blot analysis with the reduction sensitive monoclonal antibody 4H9/19 [65].

Antigens were coated onto ELISA plates (Costar, Cambridge, MA, U.S.A.) at 1 µg/ml in 100 µl PBS and incubated overnight at 4°C [21], [22], [66]. Plates were washed 3 times in PBS with 0.05% Tween-20 (0.05% PBS-T, Sigma Aldrich, St Louis, MO, U.S.A.) at room temperature (RT) and blocked with 200 µl 10% skim milk in 0.05% PBS-T for 2 hours at 37°C. The plates were washed 3 times in 0.05% PBS-T then heat inactivated plasma samples (100 µl volume diluted to 1/500 in PBS with 0.05% sodium azide) were added and incubated at RT for 2 hours. Plates were washed 3 times in 0.05% PBS-T and 100 µl of HRPO-conjugated Sheep anti human IgG (Millipore, Boronia, Melbourne, Australia) diluted 1∶2500 in 0.05% PBS-T with 5% milk added and incubated at RT for 1 hour. The plates were washed 3 times in 0.05% PBS-T and 100 µl of ABTS substrate (Sigma Aldrich, St Louis, MO, U.S.A.) was incubated in each well for 15 minutes at RT. The reaction was stopped with the addition of 100 µl of 1% SDS and read at 405 nm (Tecan Genios, Grödig, Austria). A panel of samples from malaria-exposed adults acted as positive controls to enable comparison of antibody levels between plates and different dates of experiments. A panel of serum from Melbourne donors acted as negative controls.

PfMSP1-19 specific IgG concentration was estimated by coating two-fold serial dilutions (1 µg/ml-0.00781 µg/ml) of purified human IgG (reagent grade; Sigma-Aldrich, St. Louis MO, U.S.A) and purified rabbit IgG (reagent grade; Sigma-Aldrich, St. Louis MO, U.S.A) onto plates; other wells were coated with recombinant PfMSP1-19. After blocking wells with 10% skim milk, two-fold serial dilutions of Mugil children's plasma (1/1000–1/32000) or rabbit sera against MSP1-19 (1/4000–1/256000) were added to separate wells coated with 1 µg/ml PfMSP1-19. PBS was added to wells coated with human and rabbit IgG. After incubation for 2 hours, wells were washed, then incubated with either anti-human or anti-rabbit IgG secondary antibody, then washed and processed as described above. The MSP1-19 specific IgG concentration among children's plasma, or rabbit antisera, was estimated by reference to the standard curve of human or rabbit IgG, respectively. Estimated MSP1-19 specific IgG levels among human samples were expressed relative to the rabbit MSP1-19 anti-sera.

### Estimating antibody avidity by ELISA

After incubating antigen-coated wells with human plasma or rabbit anti-MSP1-19 antibodies (as described above), wells were washed and then incubated with ammonium thiocyanate (Sigma-Aldrich, St. Louis MO, U.S.A) for 20 minutes at concentrations of 8.64 M, 6.91 M, 5.18 M, 4.32 M, 3.46 M, 2.16 M, 1.728 M, 1.08 M, 0.27 M, 0.135 M, 0.067 M. Wells were then washed and the assay was completed as described earlier. Primary antibodies were diluted for human (1/2000, 1/4000) and rabbit (1/20000, 1/40000) samples to yield OD values in the descending linear section of the absorbance curves. The ammonium thiocyanate concentration that resulted in a loss of 50% of maximum absorbance was estimated by fitting a line of best fit.

### Sample dialysis

Heat inactivated plasma samples (45 minutes at 56°C) were dialysed as described [67], [68]. Briefly, 200 µl of sample was added to 50 kDa cut-off Tube-O-Dialyzer dialysis tubes (Chemicon International, Temecula, CA, U.S.A) and the tubes were placed upside down in PBS so the dialysis membrane contacted the PBS. Samples were left to dialyse for 2 hours at 4°C and then the PBS was replaced for further dialysis overnight. Samples were centrifuged to the bottom of the dialysis tubes and transferred to 100 kDa cut-off Nanosep spin tubes (Pall Life Sciences, Ann Arbor, MI, U.S.A.) and centrifuged at 13000(G) for 30 minutes at 4°C. Sterile PBS (200 µl) was added and the spin tube centrifuged at 13000(G) at 4°C. PBS was added to bring the samples back to the original sample volume (200 µl) and stored at −70°C.

### Purification of immunoglobulins by ammonium sulfate precipitation

Immunoglobulin from twenty pooled Mugil samples with high levels of PfMSP1-19 specific antibody measured by ELISA (OD>1.7), 5 plasma samples from PNG adults and serum from non-exposed Melbourne donors were purified using ammonium sulfate precipitation [67], [68]. Briefly, 300 µl of plasma was diluted 1∶5 in normal saline. Saturated ammonium sulfate (Sigma Aldrich, St Louis, MO, U.S.A., approximately 1 kg/L in water, autoclaved) was added drop wise to make up 40% of the total volume and then incubated on ice for 30 minutes. The solution was spun at 13000(G) for 15 minutes at 4°C before removal of the supernatant. The pellet was washed in 50% ammonium sulfate solution in water before pelleting and removal of the supernatant a second time. The pellet was resuspended in 400 µl of RPMI-HEPES and the resulting solution was dialysed according to the preceding method. Immunoglobulin was resuspended off the spin column in half the original sample volume to concentrate the sample to approximately twice the initial immunoglobulin concentration.

### PfMSP1-19 specific growth inhibition assays

The PcMEGF (containing PcMSP1-19) and PfM3′ (containing PfMSP1-19) lines [27], [54] as well as the PcPHG and PfPHG GFP fluorescent MSP1-19 transfected lines [69] were used in this study and will be referred to as simply PcMSP1-19 line or PfMSP1-19 line; all transgenic parasites were generated in isolate D10 (derived from the PNG isolate FC27). The expression of the PcMSP1-19 and PfMSP1-19 alleles by the transfected parasite lines was confirmed by Western blot and immunofluorescence assays as described in O'Donnell *et al*. 2000 [27]. Parasites were cultured as described [69], [70]. PfMSP1-19 and PcMSP1-19 transfected lines were maintained on 24 ng/ml pyrimethamine (to select for MSP1-19 transfectants, Sigma Aldrich, St Louis, MO, U.S.A.) with 5 µg/ml blasticidin-S-HCl (to select for GFP transfectants, Invitrogen, Carlsbad, CA, U.S.A.) added when culturing the double transfected fluorescent lines. One to two cycles prior to assay setup, and during the assay, drugs were excluded from cultures.

The protocol for measurement of antibody inhibition of parasite growth used in this study is modified from Persson *et al.* [67] and is described and validated in detail elsewhere [69]. Parasites were cultured for one or two cycles of asexual replication. Mid to late trophozoite stage cultures (30–40 hours) were washed in fresh culture medium (37°C) and pelleted by centrifugation then diluted out to 0.5% to 1% parasitemia at 1% hematocrit for a 1 cycle assay or 0.1% to 0.3% parasitemia at 1% hematocrit for a 2 cycle assay. Fresh culture medium and fresh RBCs from 2 different donors warmed to 37°C were used during assay set-up. Parasitised RBCs at the appropriate parasitemia, at a volume of 25 µl or 50 µl, were added to a 96 well U-bottom plate (Falcon, Becton-Dickinson, Franklin Lakes, NJ, U.S.A) with 2.5 µl to 5 µl of sample (for 25 µl to 50 µl assays respectively) added to give a 1 in 10 dilution of sample. All samples were run in duplicate and in most cases in repeat experiments. A panel of non-immune serum from Melbourne donors (provided by the Australian Red Cross) were included as non-inhibitory controls. The 96 well plates were placed in a humidified air tight culture box and left at 37°C in 1% O_2_, 4% CO_2_ and 95% N_2_.

One cycle assays with measurement of parasitemia at ring-stage by microscopy and flow cytometry were cultured for approximately 24 hours until young to mid rings were present and no schizont stage parasites remained. For assessment of parasitemia by microscopy, thin smears of the cell pellets were methanol fixed and stained with 5% Giemsa (Merck, Darmstadt, Germany). Slides were blinded and one thousand RBCs were counted for each slide. Only early to late rings (0 to 20 hours old) were counted as parasitised RBCs, with each infected RBC counted as a single parasitized RBC regardless of how many parasites were in the cell. Due to the variability inherent in microscopy based counts of parasitemia [67], [69], parasitemia counts between duplicate wells were reviewed by a second person and when duplicate wells differed by >25% slides were recounted while still blinded. Where slides were recounted, all four counts were used to calculate parasitemia.

One cycle assays with measurement of parasitemia at the pigmented trophozoite stage were cultured for 48 hours post set-up until parasites were between 30–40 hours post invasion when they were prepared for flow cytometric analysis as appropriate. Two cycle trophozoite stage assays were supplemented at 48 hours post set-up (parasites 30–40 hours post invasion) with the addition of 5 µl to 10 µl (25 µl or 50 µl assay respectively) of fresh culture medium warmed to 37°C. Forty-eight (1 cycle assay) or 96 (2 cycle assay) hours post assay set-up, trophozoite stage assays were prepared for flow cytometry analysis as appropriate. For non-fluorescent parasite lines, RBCs were resuspended in 100 µl of PBS with 10 µg/ml ethidium bromide (EtBr, Bio-Rad, Hercules, CA U.S.A.), then stained for 1 hour before centrifugation, removal of the supernatant and resuspension in 200 µl of PBS. GFP fluorescent parasite lines were resuspended in PBS to make a final volume of 200 µl. Parasitemia was measured on a Becton Dickinson FACSCalibur (Franklin Lakes, NJ, U.S.A) flow cytometer with a 96 well plate sampler attached using Fl-2 (excitation wavelength 488 nm) for EtBr stained parasites and Fl-1 (excitation wavelength 488 nm) for GFP parasites. Typically, 50,000 to 80,000 RBCs were counted for each well. Samples were analysed using FlowJo software (Tree Star Inc, Ashland, OR, U.S.A).

PfMSP1-19 growth inhibitory activity measured in this assay may include both invasion inhibition and intraerythrocytic parasite growth inhibition. Although PfMSP1-19 specific inhibitory antibodies could be acting through either or both mechanisms, the two mechanisms are not differentiated in this study. For both the PcMSP1-19 and PfMSP1-19 transfected lines, parasite growth (G) was determined by dividing the mean parasitemia of duplicate wells for a test sample (S_mean_) by the mean parasitemia of 2 or more wells of non-inhibitory control (C_mean_, typically Melbourne control serum) and multiplying by 100 (G = {S_mean_/C_mea_}*100). PfMSP1-19 specific inhibitory activity (Inh_Pf_) of an individual sample was then determined by subtracting growth of the PfMSP1-19 test line (G_pf_) from growth of the PcMSP1-19 control line (G_pc_) for each sample tested (Inh_Pf_ = G_pc_-G_pf_). Rabbit antibodies to confirm MSP1-19 specific inhibition were raised against C-terminal fragments of *P. falciparum* and *P. chabaudi* MSP1-19 in an earlier study [54].

Variation in the assay was estimated as (+/−) 2 standard deviations around the mean of the Melbourne control sera used in assays. The level of potential PfMSP1-19 specific inhibition for Melbourne control samples was determined by subtracting parasite growth of the PfMSP1-19 line from the growth of the PcMSP1-19 line for each individual sample. Any difference in growth can be attributed to assay variation since there was no MSP1-19 antibodies detected in the Melbourne control sera. Two standard deviations of MSP1-19 specific inhibition for Melbourne control sera was determined separately for each set of experimental conditions and is termed the “variation threshold”. Alternatively, the number of samples above arbitrary cutoffs of 10% and 15% are also considered for 2 cycle assays using non-dialysed and dialysed plasma as has been done in other studies [58], [59].

### Statistical Analysis

Differences between categorical variables was assessed using chi-squared or Fisher's exact test, where appropriate, and differences in medians were assessed using the Mann-Whitney U test. Spearman's correlation was used to determine correlations between two data sets. These tests were performed with Prism 5.0a (GraphPad Software Inc, San Diego, CA, U.S.A.). To determine the association between antibody levels and risk of *P. falciparum* infection and symptomatic malaria, ELISA data were stratified into 3 equal groups (tertiles) reflecting low, medium and high responders according to OD values for each antigen. GIA data was categorised into (≤0%, <5%, 5–9.99% and ≥10% inhibition). Kaplan-Meier curves were generated for time-to-event (infection or clinical episode) data and compared using a log-rank test. Cox proportional hazards models were used to calculate hazard ratios for the association of antibody variables with risk of high density *P. falciparum* parasitemia and symptomatic *P. falciparum* malaria. Assumptions of proportional hazards were assessed graphically and for antibody variables that showed non-proportional hazards, results from Cox regression including the antibody variable and antibody variable by time interaction are reported [21], [22]. Hazard ratios reported are unadjusted or adjusted for the predefined covariates of age (binary variable: <9 years, ≥9 years) and location of residence [21], [22], [62]. Data analysis was performed using STATA 9.2 statistical analysis software (STATA Corporation, College Station, TX, U.S.A.).

## Results

### Acquisition of *P. falciparum* MSP1-19 specific antibodies in plasma by ELISA

Previously we have described the presence of antibodies to recombinant PfMSP1-19 (his-tagged, expressed in *E. coli*) in this cohort [43]. In the present study, antibodies to recombinant MSP1-19 from *P. falciparum* as well as *P. chabaudi* were measured. Comparison between IgG levels to the PcMSP1-19 and PfMSP1-19 antigens allowed the measurement of antibodies specific to PfMSP1-19, accounting for cross-reactive or non-specific antibodies. PfMSP1-19 specific antibodies were calculated by deducting the reactivity to PcMSP1-19 from the reactivity to PfMSP1-19 for each sample. Quantifying the relative levels of antibodies to *P. falciparum* and *P. chabaudi* MSP1-19 was also important to interpret results from growth inhibition assays using the MSP1-19 transgenic parasites, which are described later.

**Figure 1:**
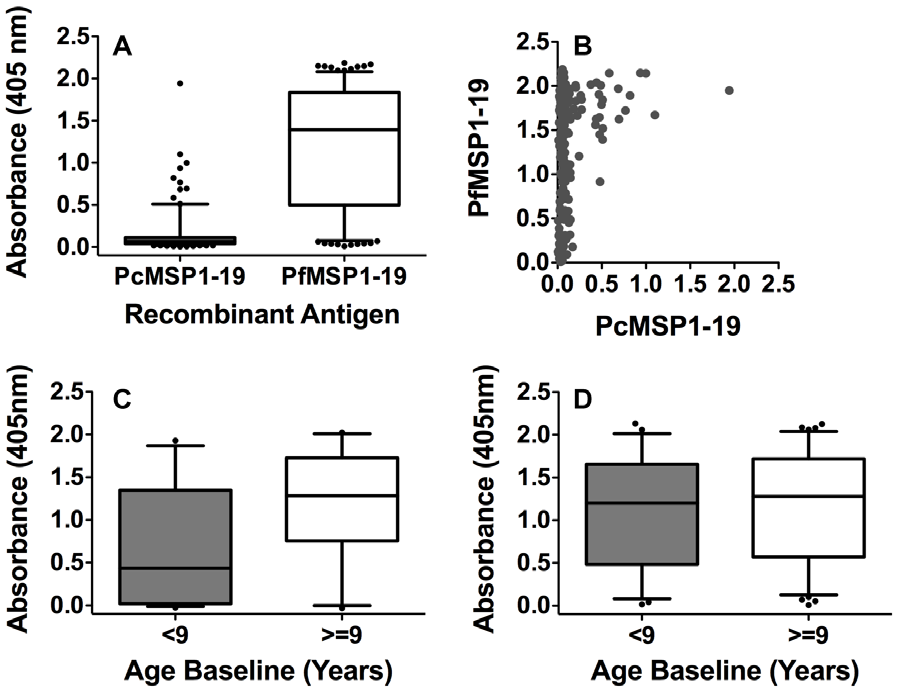
MSP1-19 IgG levels measured by ELISA and associations with age and parasitemic status at baseline bleed. (**A**) IgG reactivity against recombinant PcMSP1-19 and PfMSP1-19 for 206 Mugil plasma samples as measured by ELISA. (**B**) Correlation between levels of PcMSP1-19 and PfMSP1-19 IgG reactivity measured by ELISA for individual Mugil plasma. Levels of PfMSP1-19 specific IgG (PfMSP1-19 absorbance at 405 nm *minus* PcMSP1-19 absorbance at 405 nm) for children <9 years or ≥9 years of age (Median 9.3 years, 41% of children <9 years) at the baseline bleed separated based on (**C**) *P. falciparum* negative (n = 67) or (**D**) *P. falciparum* positive (n = 139) infection status identified by PCR at the baseline bleed. For A, C, and D, the bar and box represent median and IQR and whiskers represent the 5^th^ and 95^th^ percentiles.

Plasma antibodies were measured by ELISA to recombinant MSP1-19 from *P. falciparum* and *P. chabaudi* among the 206 baseline samples of the Mugil cohort. The median OD of the 206 Mugil children's samples for the PcMSP-19 antigen was 21-fold lower than for the PfMSP1-19 antigen (Fig. 1A) and the majority of samples showed little reactivity to the PcMSP1-19 antigen, regardless of the reactivity to the PfMSP1-19 antigen (Fig. 1B). Of the 206 samples, 192 (93.2%) were positive (defined as OD>mean +3 standard deviations of 9 non-malaria exposed Melbourne control serum) for PfMSP1-19 specific IgG. The prevalence of PfMSP1-19 antibodies was associated with age, with a significantly greater proportion of children older than 9 years (97.4%) having PfMSP1-19 IgG than children 9 years of age or less (87.9%, P = 0.007). Children greater than 9 years also had a significantly higher median OD (1.28 [0.65–1.72]) than children 9 years of age or less (0.96 [0.17–1.62], P = 0.006). A significantly higher proportion of children with concurrent parasitemia at the time of sampling had PfMSP1-19 specific IgG (98.6%) than those who were PCR negative (82.1%, P<0.0001), and the median OD of parasitemic children (1.26 [0.57–1.69]) was also significantly higher than that of PCR negative children (0.99 [0.13–1.46], P = 0.01). The associations between age or infection status and prevalence or levels of PfMSP1-19 IgG measured by ELISA were found to be largely due to the lower IgG levels in children that were aparasitemic and of 9 years of age or less at baseline bleed (Fig. 1C and 1D).

### PfMSP1-19 is not a major target of growth-inhibitory antibodies

**Figure 2:**
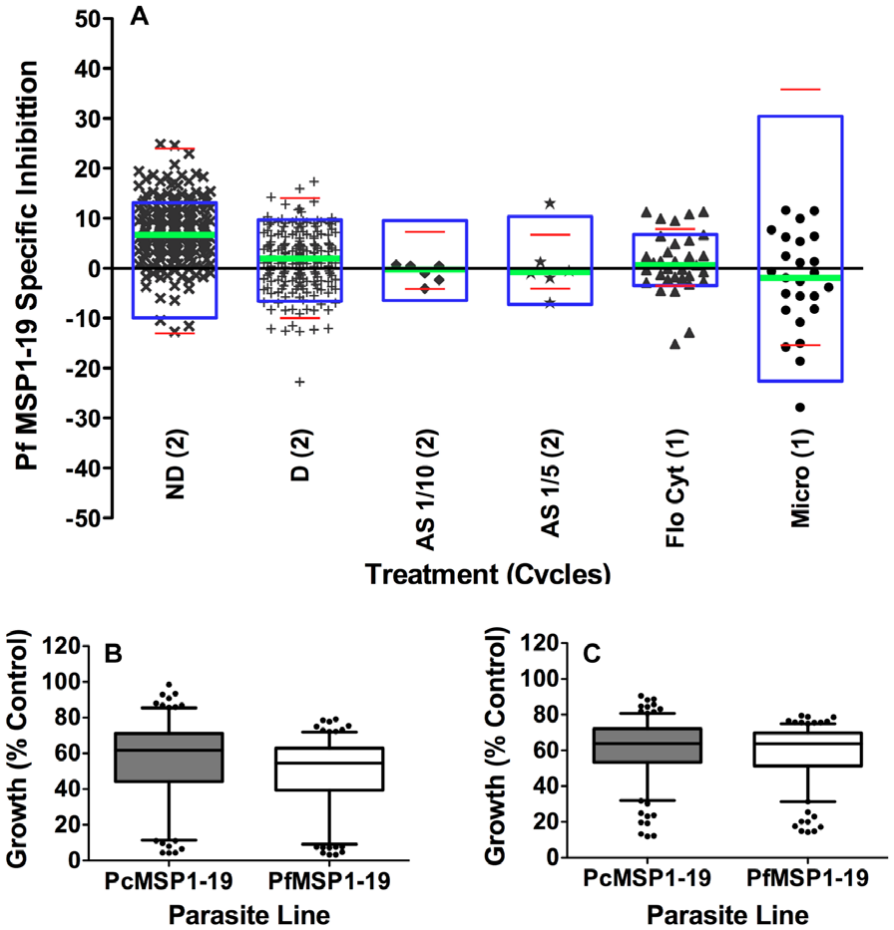
PfMSP1-19 inhibition by Mugil plasma under various experimental conditions. (**A**) Level of PfMSP1-19 specific inhibition measured by flow cytometry for non-dialysed Mugil plasma (×, n = 192, 2 cycle), dialysed Mugil plasma (+, n = 205, 2 cycle), ammonium sulfate precipitated PNG plasma at a 1 in 10 dilution (♦, n = 6, 2 cycle), ammonium sulfate precipitated PNG plasma at a 1 in 5 dilution (★, n = 6, 2 cycle), non-dialysed Mugil plasma (▴, n = 33, 1 cycle) and non-dialysed Mugil plasma measured by microscopy (•, n = 25, 1 cycle). Central bar (green) represents the median MSP1-19 specific inhibition for the plasma samples. Boxes (blue) represent 2 standard deviations of the mean MSP1-19 specific inhibition of Melbourne control samples as a measure of assay variability. Bars (red) represent the maximum and minimum values of MSP1-19 specific inhibition for Melbourne controls. PfMSP1-19 specific inhibition was defined as the growth of PcMSP1-19 line minus growth of PfMSP1-19 line, expressed as a percentage of non-inhibitory controls. Samples were tested in duplicate and correlations between duplicate experiments are outlined in Table 1. Median growth of the PcMSP1-19 line and PfMSP1-19 line in the presence of (**B**) non-dialysed Mugil plasma (n = 192) and (**C**) dialysed Mugil plasma (n = 205) in 2 cycle flow cytometry based assays. Error bars represent inter-quartile range with dots representing samples with inhibition that falls outside this range. Horizontal bar and box represent median and IQR, whiskers represent the 5^th^ and 95^th^ percentiles.

**Figure 3:**
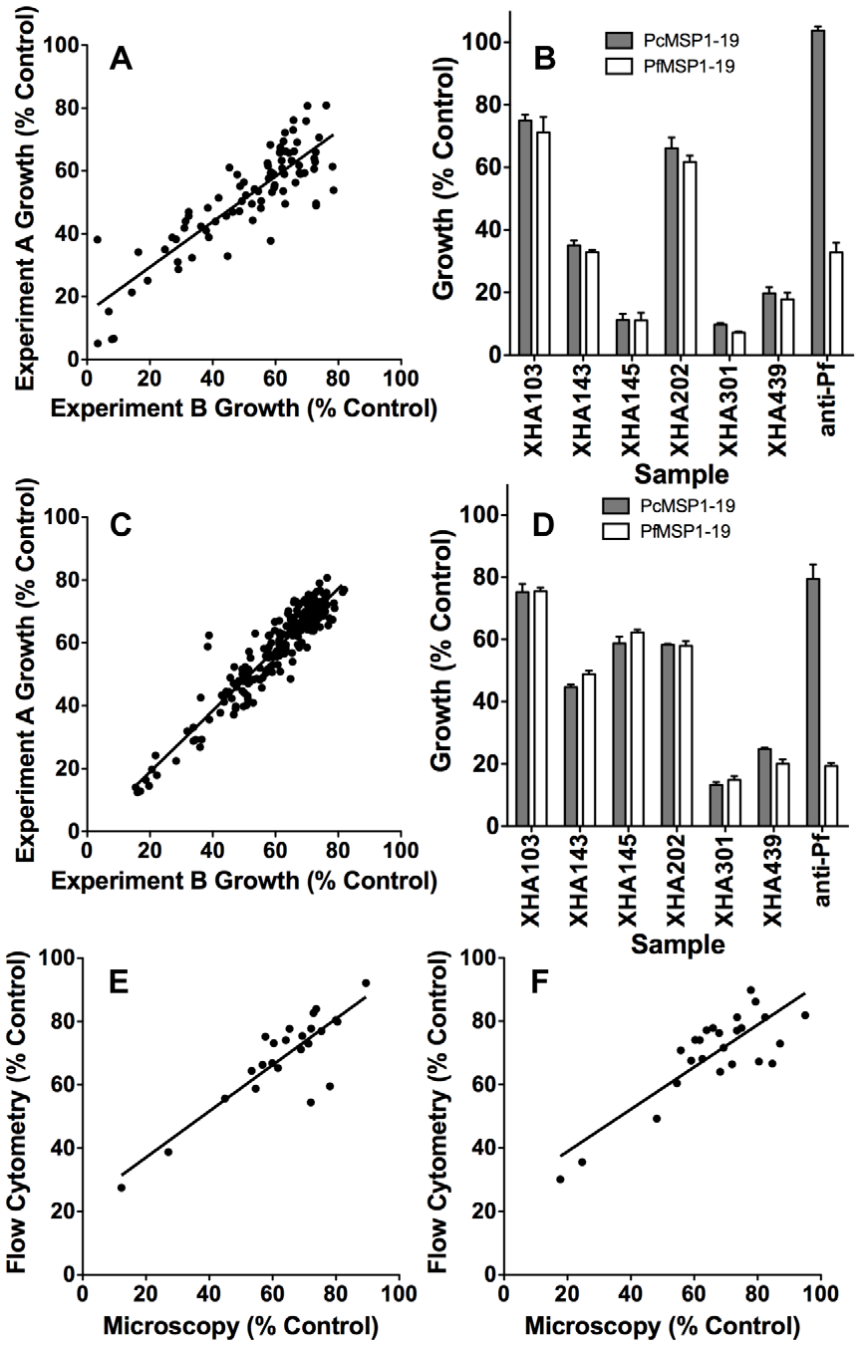
Reproducibility of PfMSP1-19 specific growth inhibition assays and levels of PfMSP1-19 specific inhibition for representative samples. (**A**) Correlation of PfMSP1-19 line growth between duplicate experiments for 85 samples of the non-dialysed Mugil plasma samples showing a high level of assay reproducibility (r_s_ = 0.88, n = 85, P<0.0001). (**B**) Subset of non-dialysed Mugil plasma samples showing total growth inhibitory activity but little or no PfMSP1-19 specific inhibitory activity in 2 cycle assays. Confirmation that the assay was able to measure PfMSP1-19 specific inhibition was provided through the use of inhibitory rabbit antisera raised against PfMSP1-19 (anti-Pf). (**C**) Correlation of PfMSP1-19 line growth between duplicate experiments for dialysed Mugil plasma samples showing a high level of assay reproducibility (r_s_ = 0.93, n = 204, P<0.0001). (**D**) Subset of dialysed Mugil plasma showing total growth inhibitory activity but little or no PfMSP1-19 specific inhibitory activity in 2 cycle assays. Correlation between parasite growth measured in flow cytometry or microscopy based 1 cycle assays for (**E**) the PcMSP1-19 line (r_s_ = 0.72, n = 25, P<0.0001) and (**F**) the PfMSP1-19 line (r_s_ = 0.59, n = 25, P = 0.0018) highlight comparable levels of growth between the two methods. Parasite growth was expressed as a percentage of the mean growth of a panel of non-dialysed or dialysed Melbourne control sera included on each assay plate.

Plasma samples, with and without dialysis, were tested in growth inhibition assays conducted over 2 cycles of parasite replication, which have been previously optimized and validated [67], [69]. Final parasitemia was measured by flow cytometry, which has a greater reproducibility than microscopy [67], [69]. Samples from 192 of the 206 children were first tested in single or duplicate experiments without prior dialysis (Fig. 2A). Results for 14 samples were excluded because of technical reasons. Comparison of median growth for the PcMSP1-19 line (expressing the C-terminal 19 kDa fragment from the rodent malaria *P. chabaudi*) and the PfMSP1-19 line (expressing the wild-type C-terminal 19 kDa fragment from *P. falciparum* D10) for all samples showed potentially specific inhibition of the PfMSP1-19 line of 7.1% (PcMSP1-19 median growth 61.7%, PfMSP1-19 median growth 54.6%; n = 192, P<0.0001; range −12.7% to 24.9%, Fig. 2B). There was evidence of PfMSP1-19 specific inhibition (i.e. growth of the PfMSP1-19 line<growth of the PcMSP1-19 line) for 61 samples when using a cut-off of >10% difference in inhibition of the two lines, and for 20 samples when using a cut-off of >15% difference. By comparison, potential PcMSP1-19 specific inhibition (i.e. growth of the PcMSP1-19 line<growth of the PfMSP1-19 line) was seen with only 3 samples when using a cut-off of >10% We further examined the significance of MSP1-19 as a target of inhibitory antibodies among those samples that demonstrated the highest levels of total growth inhibition. When examining the top third of samples with the highest level of total growth inhibition, the median PfMSP1-19 specific inhibition for these samples was only 4.1% suggesting that MSP1-19 is not a major target of acquired inhibitory antibodies. Measurement of parasite growth was highly reproducible when compared between duplicate experiments (n = 85, Table 1, Fig. 3A) with very few samples showing PfMSP1-19 specific inhibition (Fig. 3B).

**Table 1:** Reproducibility of parasite growth measurements between repeat experiments.

Cycle	Method	Plasma	Line	r_s_	n	P	Line	r_s_	n	P
2	***F***	***ND***	Pc	0.88	85	<0.0001	Pf	0.8	85	<0.0001
2	***F***	***D***	Pc	0.93	205	<0.0001	Pf	0.9	204	<0.0001
1	***F***	***ND***	Pc	0.75	25	<0.0001	Pf	0.76	25	<0.0001
1	***M***	***ND***	Pc	0.71	25	<0.0001	Pf	0.48	25	= 0.0153

***F*** = flow cytometry, ***M*** = microscopy.

***ND*** = non-dialysed plasma, ***D*** = dialysed plasma.

Pc = *P. chabaudi* MSP1-19 line; Pf = *P. falciparum* MSP1-19 line.

r_s_ = spearmans correlation coefficient.

n = number of samples compared between 2 experiments.

P = significance of correlation.

Samples were dialysed to remove anti-malarial drugs and non-specific malarial inhibitors [67] and tested in 2 cycle flow-cytometry based assays (n = 205; one sample was not dialysed due to limited volume, Fig. 2A). All dialysed plasma samples (n = 205) were tested in duplicate in two separate assays for both the PcMSP1-19 and PfMSP1-19 lines. Comparison between the median parasite growth for duplicate experiments of the PcPHG and PfPHG transfected lines indicated little or no difference in the inhibition of the two lines (Fig. 2C); only 0.1% of the growth inhibitory activity could be classified as PfMSP1-19 specific (median growth of PcMSP1-19 63.8%, PfMSP1-19 63.7%; n = 205, P = 0.2, range −22.8% to 17.4%). There was evidence of PfMSP1-19 specific inhibition for 10 samples when using a cut-off of >10% difference in inhibition of the two lines, and for only 2 samples when using a cut-off of >15% difference. By comparison, potential PcMSP1-19 specific was seen with 7 samples when using a cut-off of >10%, and one sample when using a cut-off of >15% difference. There was a high level of reproducibility between duplicate experiments (Table 1, Fig. 3C). Total parasite growth inhibition was evident for a proportion of dialysed samples; however, these inhibitory samples did not show evidence of PfMSP1-19 specific inhibition (Fig. 3D). When examining the third of samples with the highest level of total growth inhibition, the median PfMSP1-19 specific inhibition for these samples was 1.8%, further suggesting that MSP1-19 is not a major target of acquired inhibitory antibodies.

A subset of samples (n = 20) with high levels of MSP1-19 antibodies by ELISA were pooled and IgG was purified using ammonium sulfate precipitation, allowing concentration of immunoglobulin and removal of non-specific inhibitors (Fig. 2A). Plasma from 5 PNG adults with high levels of PfMSP1-19 IgG by ELISA were treated by ammonium sulfate precipitation to purify immunoglobulins. In duplicate experiments, only a low level of PfMSP1-19 specific inhibitory activity was detected for the purified IgG at a 1 in 10 dilution (median growth was 75.2% for PcMSP1-19 and 74.6% for PfMSP1-19; n = 6; range −4% to 0.7%) or using a 1 in 5 dilution (median growth was 93.1% for PcMSP1-19 and 86.9% for PfMSP1-19; n = 6; range −7% to 13%). The difference between growth of the PcMSP1-19 and PfMSP1-19 parasite lines for the ammonium sulfate precipitated children's pooled plasma was 0.6% at a 1 in 10 dilution and 6.2% at a 1 in 5 dilution.

These data suggested that PfMSP1-19 specific antibodies were not a major component of the *in vitro* growth inhibitory activity of children's plasma samples, and that there was little specific inhibition observed for the few adult samples tested. Importantly, PfMSP1-19 and PcMSP1-19 specific rabbit anti-sera tested in parallel in all assays consistently inhibited the growth of the respective lines, indicating that MSP1-19 specific inhibition could be effectively measured in these experiments (Fig. 3B and 3D).

### Further evaluation of MSP1-19 specific inhibitory antibodies by microscopy and flow cytometry

To further test for the presence of PfMSP1-19 specific inhibitory antibodies, one-cycle assays were performed to enable comparison with earlier published studies, which generally tested inhibition over one cycle only. Similar to findings with two-cycle assays, there was very little difference between the median growth of the PcMSP1-19 and PfMSP1-19 parasite lines in the presence of non-dialysed plasma samples (Fig. 2A); results suggest total PfMSP1-19 specific inhibition of only 1.1% (median growth of PcMSP1-19 75.2%, and PfMSP1-19 74.1%; n = 33, P = 0.9, range −15.1% to 11.3%). There was a high level of reproducibility between experiments (Table 1).

Since most previous studies that have measured PfMSP1-19 specific inhibitory antibodies have used microscopy to measure parasite growth, it was possible that the use of flow cytometry in the current study may have explained the failure to detect growth inhibition effectively. Therefore, 25 plasma samples were tested for PfMSP1-19 specific inhibitory activity and growth was measured by microscopy in parallel with flow cytometry. Microscopy slides were blinded by a third person for all experiments and then read by one of the authors. Counts for 2 separate microscopy experiments were available for all 25 samples with a strong correlation evident for repeat experiments (Fig. 2A, Table 1). In these assays there was little evidence of PfMSP1-19 specific growth inhibition (1.3% overall) for Mugil samples tested in a 1 cycle microscopy assay (median growth for PcMSP1-19 69.5% and PfMSP1-19 68.2%; n = 25, P = 0.5, range −27.9% to 11.6%). There was a strong correlation for measurements of PcMSP1-19 line (r_s_ = 0.72, n = 25, P<0.0001, Fig. 3E) and PfMSP1-19 line (r_s_ = 0.59, n = 25, P = 0.0018, Fig. 3F) growth between flow cytometry and microscopy based counts for the 25 Mugil plasma samples available for comparison in 1 cycle assays.

Taken together, these data suggest that the detection of PfMSP1-19 specific growth inhibitory activity was not enhanced by the use of one-cycle assays or microscopy, and confirmed findings of earlier experiments that PfMSP1-19 specific antibodies were not a major component of the *in vitro* growth inhibitory activity of children's plasma. PfMSP1-19 and PcMSP1-19 specific rabbit anti-sera inhibited growth of the target lines consistently, indicating that failure to detect MSP1-19 specific growth inhibitory activity in the Mugil plasma was not attributable to problems with the assay.

### Associations between acquired antibodies to PfMSP1-19 and protection from malaria and reinfection

**Figure 4:**
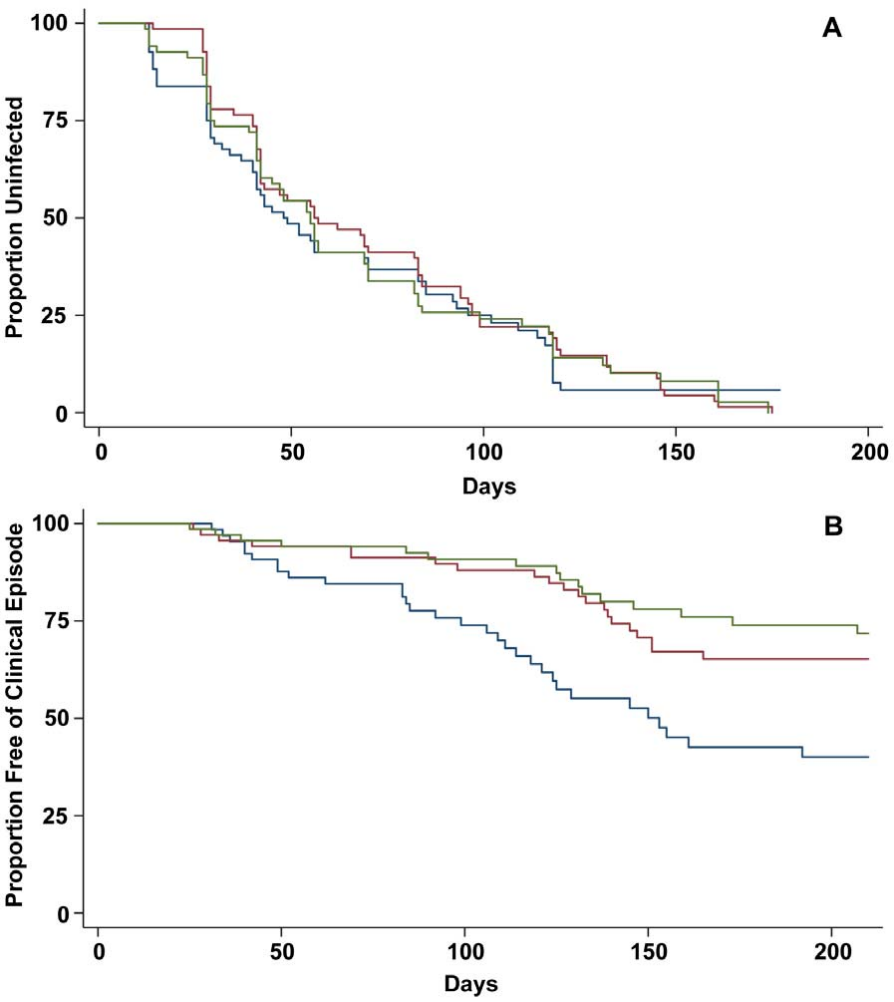
PfMSP1-19 specific IgG levels measured by ELISA and association with protection from malaria or re-infection. (**A**) Kaplan-Meier curve showing the association between PfMSP1-19 specific (PfMSP1-19 *minus* PcMSP1-19) IgG by ELISA and time to reinfection detected by PCR for low (blue), medium (red) and high (green) responders. (**B**) Kaplan-Meier curve showing the association between low, medium and high levels of PfMSP1-19 specific IgG and risk symptomatic malarial episode (fever+Pf>5000/µl).

To investigate the association between PfMSP1-19 specific IgG levels by ELISA and protection against malaria or infection, children were defined as low, medium or high responders to the PfMSP1-19 antigen (based on tertiles). Levels of response were then related to four *P. falciparum* outcomes: symptomatic *P. falciparum* malaria (defined as fever plus Pf>5000/µl); high density parasitemia (Pf>5000/µl); moderate density parasitemia (Pf>500/µl) and reinfection (detected by both light microscopy and PCR). Specific IgG to PfMSP1-19 was defined as the reactivity to recombinant PfMSP1-19 minus the reactivity to recombinant PcMSP1-19 for each sample. There was strong evidence of a protective effect of high levels of anti-PfMSP1-19 specific antibodies measured by ELISA (IgG reactivity to PfMSP1-19 minus IgG reactivity to PcMSP1-19) with protection against high density parasitemia and symptomatic *P. falciparum* infection. High levels were associated with reduced risk of high density infection (P = 0.0007; Hazard ratio [HR] = 0.40; adjusted HR = 0.48) and symptomatic *P. falciparum* infection (P = 0.0004; HR = 0.35; aHR = 0.43), Fig. 4B, Table 2). There was no evidence of an association between *P. chabaudi* MSP1-19 specific responses and high density parasitemia and symptomatic *P. falciparum* infection. In comparison to other antibody associations evaluated in this cohort, the strength of the protective association for PfMSP1-19-specific antibodies was similar to AMA1 (aHR, 0.34), but not as strong as found for antibodies to some EBA and PfRh antigens (Eg. aHR for PfRh2_2030 was 0.2; aHR for EBA175 region III–V was 0.27 [21], [22]. As reported previously for IgG MSP1-19 [43], and for other antigens [21] [22], there was no association between low, medium or high levels of PfMSP1-19 specific IgG and time to *P. falciparum* reinfection *per se* (P = 0.81 and P = 0.91, for light microscopy and PCR respectively, Fig. 4A); there was some evidence of an association between high levels of PfMSP1-19 IgG and reduced time to moderate density *P. falciparum* (>500/µl, P = 0.028).

**Table 2:** Cox regression analysis of the association of MSP1-19 responses with symptomatic and high density parasitemia.

	Fever+Pf>5000							
	HR^1^	[95%CI]		*P*	aHR^2^	[95%CI]		*P*
***Pf - Pc ELISA***								
MSP1-19 (L)^3^	1				1			
MSP1-19 (M)	0.45	0.26	0.78	0.005	0.56	0.31	1.00	0.052
MSP1-19 (H)	0.35	0.19	0.64	0.001	0.43	0.23	0.80	0.008
***Pc ELISA***								
MSP1-19 (L)	1				1			
MSP1-19 (M)	1.09	0.48	2.48	0.832	1.14	0.50	2.59	0.762
MSP1-19 (H)	0.58	0.23	1.50	0.262	0.64	0.25	1.66	0.358
***GIA dialysed***								
MSP1-19 (0%)^4^	1				1			
MSP1-19 (<5%)	1.85	0.98	3.52	0.059	1.72	0.90	3.29	0.104
MSP1-19 (5–9.9%)	2.68	1.37	5.24	0.004	2.71	1.33	5.52	0.006
MSP1-19 (>10%)	10.41	4.36	24.84	<0.001	8.20	3.34	20.11	<0.001
***GIA non-dialysed***								
MSP1-19 (0%)	1				1			
MSP1-19 (<5%)	0.67	0.16	2.81	0.584	0.89	0.21	3.77	0.869
MSP1-19 (5–9.9%)	0.88	0.23	3.32	0.852	1.03	0.27	3.96	0.967
MSP1-19 (>10%)	1.66	0.48	5.79	0.424	1.90	0.53	6.81	0.323

1 HR – hazard ratio for risk of malaria (fever+*P. falciparum* parasitemia >5000 parasites per microlitre of blood).

2 aHR – adjusted hazard ratios (adjusted for age and location of residence).

3 High (H), medium (M), and low (L) antibody levels were defined by tertiles.

4 Level of MSP1-19 specific inhibition is indicated in brackets.

In order to assess the association of growth inhibitory antibodies with protection against clinical malaria, MSP1-19 *P. falciparum*-specific growth inhibition data from testing both dialysed and non-dialysed samples was used. Although there was a strong association between antibodies to PfMSP1-19 measured by ELISA and protective immunity, there was no evidence that PfMSP1-19 specific inhibitory antibodies were associated with protection from malaria. Individuals with potentially significant levels of PfMSP1-19 specific inhibition (>10%) using dialysed plasma had an increased risk of symptomatic and high density parasitemia compared to those with no inhibition (Table 2). However, since there was little evidence of PfMSP1-19 specific growth inhibition in samples from the cohort, evaluating associations between growth inhibition and protection from malaria are problematic. There was no association of PfMSP1-19 specific growth inhibition and risk of symptomatic malaria for non-dialysed samples (Table 2).

### Concentration and avidity of PfMSP1-19 antibodies estimated by ELISA

**Figure 5:**
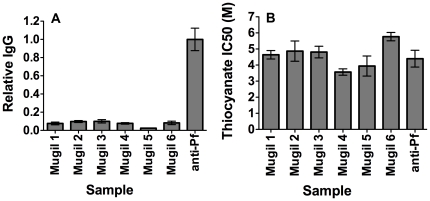
PfMSP1-19 antibody concentration and avidity of binding in Mugil plasma and rabbit sera. (**A**) Comparison of PfMSP1-19 specific antibody levels estimated by ELISA between Mugil plasma and growth inhibitory vaccinated rabbit sera raised against PfMSP1-19 (anti-Pf). Relative levels are expressed as a proportion of the PfMSP1-19 rabbit antisera. (**B**) Comparison of IC50 (M) for ammonium thiocyanate inhibition of PfMSP1-19 antibody binding to recombinant PfMSP1-19 for Mugil plasma and growth inhibitory vaccinated rabbit sera raised against PfMSP1-19. The 6 Mugil plasma samples tested were selected based on having high levels of PfMSP1-19 IgG as measured by ELISA. Bars represent the mean and range of 2 independent experiments (antibody concentration in Figure A) or the mean and standard deviation of three independent experiments (thiocyanate concentration in Figure B).

To assess whether the lack of MSP1-19 specific growth inhibitory activity could be attributed to low PfMSP1-19 antibody concentration in children's samples or low antibody avidity or affinity, PfMSP1-19 IgG levels and avidity were compared between growth inhibitory rabbit antisera and a subset of children's plasma samples; a selection of plasma samples with high reactivity to MSP1-19 by ELISA was used. PfMSP1-19 specific antibody levels in growth inhibitory rabbit antisera that was generated by immunization was estimated to be 12.5-fold higher than that of the panel of highly reactive Mugil plasma samples (n = 6, Fig. 5A). The rabbit antisera routinely caused around 70% growth inhibition at 1/10 dilution in 2-cycle assays (Fig. 3B), with dilution to 1/80 resulting in a loss of PfMSP1-19 specific growth inhibitory activity (data not shown). At a 1 in 80 dilution, the concentration of PfMSP1-19 specific IgG in rabbit antisera would still be approximately 1.5 fold higher than the median concentration for the children's plasma samples tested.

The avidity of PfMSP1-19 specific antibody binding to recombinant PfMSP1-19 was estimated using serial dilutions of ammonium thiocyanate, added to wells after first allowing antibodies to bind immobilized antigen. The concentration of ammonium thiocyanate that reduced OD by 50%, compared to control (no ammonium thiocyanate added) was used as a measure to compare avidity between samples. The concentration required for a 50% reduction for the rabbit antisera (4.4 M) was comparable to that observed for a panel of Mugil plasma samples (median 4.7 M, range 3.6 M to 5.8 M, n = 6, Fig. 5B). We also calculated the relative reduction in antibody binding by thiocyanate treatment among samples. At 4.3 M, ammonium thiocyanate reduced antibody binding among children's by 49.5% (median; range 29.8–82%) and by 50.5% for the rabbit antisera (all sera tested at 1/2000 dilution). Together, these data suggest that the lack of MSP1-19 specific growth-inhibitory activity of children's plasma samples compared to rabbit antisera could be explained by a lower concentration of specific antibodies among children's plasma samples, and not by lower avidity of antibody binding.

## Discussion

MSP1-19 is an important antigenic region of the larger 42 kDa fragment of MSP1, which has long been considered a promising *P. falciparum* vaccine candidate. Several ELISA based studies have reported significant associations between levels of antibodies to MSP1-19 and protection from malaria [38], [40]–[42], [71]–[74]; a recent meta-analysis indicated a 19% reduction in malaria risk for individuals with MSP1-19 antibodies [20]. Functional growth inhibitory MSP1-19 specific antibodies have also been reported in samples from malaria exposed individuals using *in vitro* assays [54]–[61]. To date, no study has reported associations between PfMSP1-19 specific growth inhibitory activity and protection from symptomatic malaria, or quantified the importance of MSP1-19 as a target of the overall growth-inhibitory activity of acquired human antibodies. Here we report that although IgG to MSP1-19 measured by ELISA is associated with protection from malaria, MSP1-19 is not a significant target of growth-inhibitory antibodies in this cohort.

Of the 206 Mugil plasma samples collected at baseline, 95.6% were found to be PfMSP1-19 IgG positive by ELISA, suggesting the majority of children had had significant prior exposure to *P. falciparum* MSP1-19 antigen, consistent with the high rate of *P. falciparum* reinfections (96% measured by PCR) observed in the study [62]. Older children, and children that were PCR positive for *P. falciparum* infection at baseline, were found to have higher levels of PfMSP1-19 antibodies measured by ELISA than younger children and children that were PCR negative. These data suggest that levels of PfMSP1-19 specific antibodies measured by ELISA are associated with recent and cumulative exposure to *P. falciparum* as expected.

Analysis of the association between PfMSP1-19 antibody levels measured by ELISA and first *P. falciparum* malaria episode post drug treatment indicated that high levels of PfMSP1-19 specific (PfMSP1-19 OD *minus* PcMSP1-19 OD) antibodies were associated with reduced risk of symptomatic malaria, which remained significant after adjusting for differences in age and location of residence. Time to first moderate or high density *P. falciparum* infection was significantly increased in children with high PfMSP1-19 antibody levels, which is consistent with the current understanding of acquired immunity and proposed mechanisms of action for anti-MSP1-19 antibodies. These findings are similar to a previous study of MSP1-19 antibodies in this cohort [43]. However, in this study we assessed IgG specificity to PfMSP1-19 by deducting reactivity to recombinant MSP1-19 of *P. chabaudi* in order to account for non-specific binding, which is a concern that is often expressed by researchers in the field.

The results of this study support the findings of a number of other studies which have shown an association between IgG antibody levels to PfMSP1-19 measured by ELISA and control of parasitemia or protection from symptomatic *P. falciparum* malaria [38], [40]–[42], [71]–[74]. The association between MSP1-19 antibody levels and protection from disease is evident in both infants and young children, which suggests that protection associated with MSP1-19 antibodies develops early in people's exposure to malaria [38], [40], [74]. Combined, these studies suggest that IgG antibodies to MSP1-19 may be an important component of immunity to *P. falciparum* malaria or alternatively may act as a strong predictive marker of exposure, and hence immunity.

Our comprehensive testing suggests that there is little PfMSP1-19 specific growth inhibition in plasma samples from the Mugil cohort, under various experimental conditions. Furthermore, any potential MSP1-19 specific inhibitory activity was not associated with protection from malaria or re-infection. The median level of PfMSP1-19 specific inhibition for non-dialysed Mugil plasma tested in a 2 cycle assay, dialysed Mugil plasma tested in a 2 cycle assay, non-dialysed Mugil plasma in a 1 cycle assay measured by flow cytometry or microscopy, and ammonium sulfate precipitated IgG at a 1 in 10 or 1 in 5 dilution was generally minimal and lower than the levels reported in other studies [54]–[58]. The highest median PfMSP1-19 specific inhibition was recorded with 2 cycle assays using non-dialysed samples. However, there were very few samples with PfMSP1-19 specific inhibition above a commonly used cut-off of 15% and no samples had levels of PfMSP1-19 specific inhibition approaching the highest levels seen in other studies, despite the fact that two cycle growth inhibition assays have been previously reported to have greater sensitivity compared to 1 cycle assays [67], [69], [75]. This suggests that the PfMSP1-19 specific inhibition seen for the two cycle non-dialysed samples might be attributable to assay variation rather than antibody-mediated growth inhibition. Other antigens have been identified or proposed as targets of acquired human growth inhibitory antibodies, including AMA1 [76], erythrocyte binding antigens [66], and PfRh ligands [66]. These may be important targets of inhibitory antibodies in this population.

Importantly, rabbit polyclonal antibodies raised against recombinant PfMSP1-19 and PcMSP1-19 were used in all assays and consistently found to specifically inhibited growth of the target parasite lines. This suggests that antibody mediated MSP1-19 specific inhibition in malaria exposed samples could be measured if the inhibitory activity of the antibodies was high enough. In this study, a variability threshold was determined from paired (PcMSP1-19 line or PfMSP1-19 line) Melbourne control samples to provide a reliable indicator of the amount of measured MSP1-19 specific inhibition that could be attributable to assay variability. In this way it was hoped to establish a more valid cut-off for defining MSP1-19 positivity or negativity than using an arbitrary value of 5 to 15% as has been adopted previously [58]–[60]. Using this method, notional MSP1-19 specific inhibition detected in the majority of samples could be explained by assay variability.

It was noticeable that the number of samples displaying PfMSP1-19 specific inhibition was usually balanced by the number of samples showing *negative* PfMSP1-19 specific inhibition (i.e. PcMSP1-19 specific inhibition), the exception being the two cycle non-dialysed data. Given the distribution of MSP1-19 specific inhibition it would appear likely that *negative* PfMSP1-19 specific inhibition reflects assay variability rather than a PcMSP1-19-like inhibitor. It is interesting to note that the maximum and minimum levels of notional MSP1-19 specific inhibition for Melbourne control samples lie at similar levels to the maximum and minimum levels of inhibition seen for the Mugil samples. Again this suggests that MSP1-19 specific inhibition detected in Mugil samples could be explained by assay variability. Importantly, the variability seen in Melbourne control samples was not an indication of poor reproducibility of flow cytometry based assays, since there was a high level of reproducibility for measurements of parasite growth between duplicate wells and between experiments across this study.

One possible explanation for the lack of PfMSP1-19 specific growth inhibition seen in this study might be related to the level of exposure for this cohort compared to others. Previous studies reported high levels of PfMSP1-19 specific inhibition in samples collected from children and adults living in highly malaria endemic areas [54], [61] with no correlation between age and PfMSP1-19 specific growth inhibition identified for one of the studies [61]. Other studies have reported PfMSP1-19 specific growth inhibitory activity in samples collected from people with limited exposure to *P. falciparum* malaria [55], [56]. Therefore, these studies suggest that previously detected PfMSP1-19 growth inhibitory antibodies are not confined to samples collected from areas of high endemicity and from people with high levels of exposure. The levels and growth inhibitory activity of PfMSP1-19 specific antibodies in plasma samples during the acute phase of reinfection have not been examined in this study; antibodies were only measured at enrolment. It is likely that total antibody levels to PfMSP1-19 would rise in response to reinfection. However, the minimal levels of PfMSP1-19 specific growth inhibitory activity seen in the study population, and the lack of any association between MSP1-19 specific inhibitory antibodies and active parasitemia at enrolment, suggests that subsequent parasite reinfection is unlikely to increase PfMSP1-19 inhibitory activity.

Estimation of PfMSP-19 IgG concentration in rabbit antisera and Mugil plasma with high levels of PfMSP1-19 antibodies suggested that antibody concentration may be an important factor limiting ability to detect PfMSP1-19 specific inhibition using this assay. PfMSP1-19 specific IgG was 12.5 fold higher in rabbit antisera than in a panel of Mugil plasma. When the rabbit antisera were diluted down such that the concentration of IgG was similar to that seen in the Mugil plasma, there was loss of growth inhibitory activity. This suggests that levels of PfMSP1-19 antibodies in Mugil samples may not be high enough to allow detection of MSP1-19 specific growth inhibition in this assay. The avidity of PfMSP1-19 specific binding to the recombinant PfMSP1-19 antigen appeared comparable in rabbit antisera and Mugil plasma, suggesting that differences in antibody avidity or affinity did not contribute to the reduced PfMSP1-19 specific inhibition by Mugil plasma compared to rabbit antisera. However, these results should be interpreted with caution since using ammonium thiocyanate elution in ELISA provides an estimate of the relative avidity of antibodies *in vitro*, but may not faithfully represent avidity or affinity *in vivo*. Additionally, differences in the fine specificity of antibodies may also contribute to differences observed in the inhibitory activity of human and rabbit antibodies.

The limited PfMSP1-19 growth inhibitory activity detected in this study differs markedly to earlier published studies reporting greater levels of MSP1-19 specific inhibition in serum or plasma (Table S1) [54], [55], [61]. One possible explanation noted by Murhandarwati *et al.* (2009) [59], is that several earlier studies used the pyrimethamine sensitive D10 wildtype line as the test line compared to the pyrimethamine resistant PcMEGF line [54], [55], [61]. In this and other recent studies [56]–[60], the pyrimethamine resistant PfM3′ (also the PfPHG line this study) PfMSP1-19 transfected line was used together with the PcMEGF or PyMEGF (also the PcPHG line this study) comparison line, ensuring that any residual pyrimethamine in plasma samples should not contribute to differential growth between the lines. More recent studies [59], [60], including this study, have also moved towards using flow cytometry based counts of parasitemia which has reduced variability and improved reproducibility compared to microscopy based measurements [67], [69], [75]. It is possible that microscopy-based assays are able to measure growth differences between the Pf and Pc lines that are not detected by flow cytometry, but our comparison of the two assays have suggested that they give similar results.

In conclusion, although MSP1-19 IgG levels measured by ELISA were associated with protection, there was little or no PfMSP1-19 specific growth inhibitory activity measurable in the Mugil cohort, probably because antibodies were not of a high enough concentration. This suggests that the association between MSP1-19 IgG levels measured by ELISA and protection from disease may be a marker of protective immunity rather than being directly involved in protective mechanisms, or may indicate that MSP1-19 antibodies contribute to protection by mechanisms other than growth inhibition. Studies in a humanized mouse model suggest that antibodies to MSP1-19 can protect against parasitemia through an interaction dependent on Fc Receptors and phagocytic cells, rather than inhibition of invasion [52]. In conclusion, the findings presented here contribute to our understanding of human immunity to malaria, particularly the targets and mechanisms of immunity, and will be valuable for informing vaccine development of MSP1 and other antigens.

## Supporting Information

Table S1 Summary of PfMSP1-19 specific inhibition levels reported in different studies.
***^a^*** ** ***S*** = serum. ***P*** = plasma.
***^b^*** ** ***M*** = microscopy based assay. ***F*** = flow cytometry based assay. ***H*** = Hypoxanthine uptake assay.
***^c^*** Microscopy based assays for Bagabag Island and Madang samples were completed using the D10 wildtype line. Hypoxanthine uptake based assays for Bagabag Island and Madang samples were completed using the transfected D10-PfM3′ line [54].
***^d^*** Interpretation from Figures 2C and 2D and information in text.
***^d^*** Inhibition minimum at 6 months (16% n = 24) maximum at 24–30 months (29%, n = 19).
***^e^*** Inhibition range for 9 of 20 returned travelers considered to have significant PfMSP1-19 specific inhibition. Data for remaining 11 travelers not reported.
***^f^*** Interpretation from Figures 2B and 3. Spaghetti plots of timepoints for all samples available in supplementary file 4 of the manuscript.
***^g^*** Calculated median for all 137 samples listed in table 1 of the manuscript. Calculated medians for the 3 timepoints are t = 0 (9%, n = 65), t = 1 (10%, n = 37), t = 28 (−14%, n = 35). NR = not reported in manuscript.
(DOCX)

## References

[1] Elliott SR, Beeson JG (2008). Estimating the burden of global mortality in children aged <5 years by pathogen-specific causes.. Clin Infect Dis.

[2] Guerra CA, Gikandi PW, Tatem AJ, Noor AM, Smith DL (2008). The limits and intensity of *Plasmodium falciparum* transmission: implications for malaria control and elimination worldwide.. PLoS Med.

[3] Rowe AK, Rowe SY, Snow RW, Korenromp EL, Schellenberg JR (2006). The burden of malaria mortality among African children in the year 2000.. Int J Epidemiol.

[4] Snow RW, Guerra CA, Noor AM, Myint HY, Hay SI (2005). The global distribution of clinical episodes of *Plasmodium falciparum* malaria.. Nature.

[5] Gaur D, Mayer DC, Miller LH (2004). Parasite ligand-host receptor interactions during invasion of erythrocytes by Plasmodium merozoites.. Int J Parasitol.

[6] Marsh K, Kinyanjui S (2006). Immune effector mechanisms in malaria.. Parasite Immunol.

[7] al-Yaman F, Genton B, Anders R, Taraika J, Ginny M (1995). Assessment of the role of the humoral response to *Plasmodium falciparum* MSP2 compared to RESA and SPf66 in protecting Papua New Guinean children from clinical malaria.. Parasite Immunol.

[8] Taylor RR, Allen SJ, Greenwood BM, Riley EM (1998). IgG3 antibodies to *Plasmodium falciparum* merozoite surface protein 2 (MSP2): increasing prevalence with age and association with clinical immunity to malaria.. Am J Trop Med Hyg.

[9] Alifrangis M, Lemnge MM, Moon R, Theisen M, Bygbjerg I (1999). IgG reactivities against recombinant Rhoptry-Associated Protein-1 (rRAP-1) are associated with mixed Plasmodium infections and protection against disease in Tanzanian children.. Parasitology.

[10] Migot-Nabias F, Luty AJ, Ringwald P, Vaillant M, Dubois B (1999). Immune responses against *Plasmodium falciparum* asexual blood-stage antigens and disease susceptibility in Gabonese and Cameroonian children.. Am J Trop Med Hyg.

[11] Oeuvray C, Theisen M, Rogier C, Trape JF, Jepsen S (2000). Cytophilic immunoglobulin responses to *Plasmodium falciparum* glutamate-rich protein are correlated with protection against clinical malaria in Dielmo, Senegal.. Infect Immun.

[12] Metzger WG, Okenu DM, Cavanagh DR, Robinson JV, Bojang KA (2003). Serum IgG3 to the *Plasmodium falciparum* merozoite surface protein 2 is strongly associated with a reduced prospective risk of malaria.. Parasite Immunol.

[13] Soe S, Theisen M, Roussilhon C, Aye KS, Druilhe P (2004). Association between protection against clinical malaria and antibodies to merozoite surface antigens in an area of hyperendemicity in Myanmar: complementarity between responses to merozoite surface protein 3 and the 220-kilodalton glutamate-rich protein.. Infect Immun.

[14] Polley SD, Mwangi T, Kocken CH, Thomas AW, Dutta S (2004). Human antibodies to recombinant protein constructs of *Plasmodium falciparum* Apical Membrane Antigen 1 (AMA1) and their associations with protection from malaria.. Vaccine.

[15] Polley SD, Tetteh KK, Lloyd JM, Akpogheneta OJ, Greenwood BM (2007). *Plasmodium falciparum* merozoite surface protein 3 is a target of allele-specific immunity and alleles are maintained by natural selection.. J Infect Dis.

[16] Osier FH, Polley SD, Mwangi T, Lowe B, Conway DJ (2007). Naturally acquired antibodies to polymorphic and conserved epitopes of *Plasmodium falciparum* merozoite surface protein 3.. Parasite Immunol.

[17] Roussilhon C, Oeuvray C, Muller-Graf C, Tall A, Rogier C (2007). Long-term clinical protection from falciparum malaria is strongly associated with IgG3 antibodies to merozoite surface protein 3.. PLoS Med.

[18] Osier FH, Fegan G, Polley SD, Murungi L, Verra F (2008). Breadth and magnitude of antibody responses to multiple *Plasmodium falciparum* merozoite antigens are associated with protection from clinical malaria.. Infect Immun.

[19] Nebie I, Diarra A, Ouedraogo A, Soulama I, Bougouma EC (2008). Humoral responses to *Plasmodium falciparum* blood-stage antigens and association with incidence of clinical malaria in children living in an area of seasonal malaria transmission in Burkina Faso, West Africa.. Infect Immun.

[20] Fowkes FJ, Richards JS, Simpson JA, Beeson JG (2010). The relationship between anti-merozoite antibodies and incidence of *Plasmodium falciparum* malaria: A systematic review and meta-analysis.. PLoS Med.

[21] Reiling L, Richards JS, Fowkes FJ, Barry AE, Triglia T (2010). Evidence that the erythrocyte invasion ligand PfRh2 is a target of protective immunity against *Plasmodium falciparum* malaria.. J Immunol.

[22] Richards JS, Stanisic DI, Fowkes FJ, Tavul L, Dabod E (2010). Association between naturally acquired antibodies to erythrocyte-binding antigens of *Plasmodium falciparum* and protection from malaria and high-density parasitemia.. Clin Infect Dis.

[23] Beeson JG, Osier FH, Engwerda CR (2008). Recent insights into humoral and cellular immune responses against malaria.. Trends Parasitol.

[24] Richards JS, Beeson JG (2009). The future for blood-stage vaccines against malaria.. Immunol Cell Biol.

[25] Tanabe K, Mackay M, Goman M, Scaife JG (1987). Allelic dimorphism in a surface antigen gene of the malaria parasite *Plasmodium falciparum*.. J Mol Biol.

[26] Miller LH, Roberts T, Shahabuddin M, McCutchan TF (1993). Analysis of sequence diversity in the *Plasmodium falciparum* merozoite surface protein-1 (MSP-1).. Mol Biochem Parasitol.

[27] O'Donnell RA, Saul A, Cowman AF, Crabb BS (2000). Functional conservation of the malaria vaccine antigen MSP-119across distantly related Plasmodium species.. Nat Med.

[28] Sanders PR, Kats LM, Drew DR, O'Donnell RA, O'Neill M (2006). A set of glycosylphosphatidyl inositol-anchored membrane proteins of *Plasmodium falciparum* is refractory to genetic deletion.. Infect Immun.

[29] Lyon JA, Geller RH, Haynes JD, Chulay JD, Weber JL (1986). Epitope map and processing scheme for the 195,000-dalton surface glycoprotein of *Plasmodium falciparum* merozoites deduced from cloned overlapping segments of the gene.. Proc Natl Acad Sci U S A.

[30] McBride JS, Heidrich HG (1987). Fragments of the polymorphic Mr 185,000 glycoprotein from the surface of isolated *Plasmodium falciparum* merozoites form an antigenic complex.. Mol Biochem Parasitol.

[31] Blackman MJ, Heidrich HG, Donachie S, McBride JS, Holder AA (1990). A single fragment of a malaria merozoite surface protein remains on the parasite during red cell invasion and is the target of invasion-inhibiting antibodies.. J Exp Med.

[32] Blackman MJ, Ling IT, Nicholls SC, Holder AA (1991). Proteolytic processing of the *Plasmodium falciparum* merozoite surface protein-1 produces a membrane-bound fragment containing two epidermal growth factor-like domains.. Mol Biochem Parasitol.

[33] Blackman MJ, Holder AA (1992). Secondary processing of the *Plasmodium falciparum* merozoite surface protein-1 (MSP1) by a calcium-dependent membrane-bound serine protease: shedding of MSP133 as a noncovalently associated complex with other fragments of the MSP1.. Mol Biochem Parasitol.

[34] Blackman MJ, Scott-Finnigan TJ, Shai S, Holder AA (1994). Antibodies inhibit the protease-mediated processing of a malaria merozoite surface protein.. J Exp Med.

[35] Boyle MJ, Wilson DW, Richards JS, Riglar DT, Tetteh KK (2010). Isolation of viable *Plasmodium falciparum* merozoites to define erythrocyte invasion events and advance vaccine and drug development.. Proc Natl Acad Sci U S A.

[36] Goel VK, Li X, Chen H, Liu SC, Chishti AH (2003). Band 3 is a host receptor binding merozoite surface protein 1 during the *Plasmodium falciparum* invasion of erythrocytes.. Proc Natl Acad Sci U S A.

[37] Boyle MJ, Richards JS, Gilson PR, Chai W, Beeson JG (2010). Interactions with heparin-like molecules during erythrocyte invasion by *Plasmodium falciparum* merozoites.. Blood.

[38] Hogh B, Marbiah NT, Burghaus PA, Andersen PK (1995). Relationship between maternally derived anti-*Plasmodium falciparum* antibodies and risk of infection and disease in infants living in an area of Liberia, west Africa, in which malaria is highly endemic.. Infect Immun.

[39] Shai S, Blackman MJ, Holder AA (1995). Epitopes in the 19 kDa fragment of the *Plasmodium falciparum* major merozoite surface protein-1 (PfMSP-1(19)) recognized by human antibodies.. Parasite Immunol.

[40] Branch OH, Udhayakumar V, Hightower AW, Oloo AJ, Hawley WA (1998). A longitudinal investigation of IgG and IgM antibody responses to the merozoite surface protein-1 19-kiloDalton domain of *Plasmodium falciparum* in pregnant women and infants: associations with febrile illness, parasitemia, and anemia.. Am J Trop Med Hyg.

[41] Conway DJ, Cavanagh DR, Tanabe K, Roper C, Mikes ZS (2000). A principal target of human immunity to malaria identified by molecular population genetic and immunological analyses.. Nat Med.

[42] Perraut R, Marrama L, Diouf B, Sokhna C, Tall A (2005). Antibodies to the conserved C-terminal domain of the *Plasmodium falciparum* merozoite surface protein 1 and to the merozoite extract and their relationship with *in vitro* inhibitory antibodies and protection against clinical malaria in a Senegalese village.. J Infect Dis.

[43] Stanisic DI, Richards JS, McCallum FJ, Michon P, King CL (2009). Immunoglobulin G subclass-specific responses against *Plasmodium falciparum* merozoite antigens are associated with control of parasitemia and protection from symptomatic illness.. Infect Immun.

[44] Egan AF, Burghaus P, Druilhe P, Holder AA, Riley EM (1999). Human antibodies to the 19 kDa C-terminal fragment of *Plasmodium falciparum* merozoite surface protein 1 inhibit parasite growth *in vitro*.. Parasite Immunol.

[45] Chappel JA, Egan AF, Riley EM, Druilhe P, Holder AA (1994). Naturally acquired human antibodies which recognize the first epidermal growth factor-like module in the *Plasmodium falciparum* merozoite surface protein 1 do not inhibit parasite growth *in vitro*.. Infect Immun.

[46] Daly TM, Long CA (1995). Humoral response to a carboxyl-terminal region of the merozoite surface protein-1 plays a predominant role in controlling blood-stage infection in rodent malaria.. J Immunol.

[47] Hirunpetcharat C, Tian JH, Kaslow DC, van Rooijen N, Kumar S (1997). Complete protective immunity induced in mice by immunization with the 19-kilodalton carboxyl-terminal fragment of the merozoite surface protein-1 (MSP1[19]) of *Plasmodium yoelli* expressed in Saccharomyces cerevisiae: correlation of protection with antigen-specific antibody titer, but not with effector CD4+ T cells.. J Immunol.

[48] Ahlborg N, Ling IT, Howard W, Holder AA, Riley EM (2002). Protective immune responses to the 42-kilodalton (kDa) region of *Plasmodium yoelli* merozoite surface protein 1 are induced by the C-terminal 19-kDa region but not by the adjacent 33-kDa region.. Infect Immun.

[49] Wipasa J, Xu H, Makobongo M, Gatton M, Stowers A (2002). Nature and specificity of the required protective immune response that develops postchallenge in mice vaccinated with the 19-kilodalton fragment of *Plasmodium yoelli* merozoite surface protein 1.. Infect Immun.

[50] Singh S, Miura K, Zhou H, Muratova O, Keegan B (2006). Immunity to recombinant *Plasmodium falciparum* merozoite surface protein 1 (MSP1): protection in Aotus nancymai monkeys strongly correlates with anti-MSP1 antibody titer and *in vitro* parasite-inhibitory activity.. Infect Immun.

[51] Nwuba RI, Sodeinde O, Anumudu CI, Omosun YO, Odaibo AB (2002). The human immune response to *Plasmodium falciparum* includes both antibodies that inhibit merozoite surface protein 1 secondary processing and blocking antibodies.. Infect Immun.

[52] McIntosh RS, Shi J, Jennings RM, Chappel JC, de Koning-Ward TF (2007). The importance of human FcgammaRI in mediating protection to malaria.. PLoS Pathog.

[53] Gilson PR, O'Donnell RA, Nebl T, Sanders PR, Wickham ME (2008). MSP1(19) miniproteins can serve as targets for invasion inhibitory antibodies in *Plasmodium falciparum* provided they contain the correct domains for cell surface trafficking.. Mol Microbiol.

[54] O'Donnell RA, de Koning-Ward TF, Burt RA, Bockarie M, Reeder JC (2001). Antibodies against merozoite surface protein (MSP)-1(19) are a major component of the invasion-inhibitory response in individuals immune to malaria.. J Exp Med.

[55] John CC, O'Donnell RA, Sumba PO, Moormann AM, de Koning-Ward TF (2004). Evidence that invasion-inhibitory antibodies specific for the 19-kDa fragment of merozoite surface protein-1 (MSP-1 19) can play a protective role against blood-stage *Plasmodium falciparum* infection in individuals in a malaria endemic area of Africa.. J Immunol.

[56] Dent A, Malhotra I, Mungai P, Muchiri E, Crabb BS (2006). Prenatal malaria immune experience affects acquisition of *Plasmodium falciparum* merozoite surface protein-1 invasion inhibitory antibodies during infancy.. J Immunol.

[57] Eisen DP, Wang L, Jouin H, Murhandarwati EE, Black CG (2007). Antibodies elicited in adults by a primary *Plasmodium falciparum* blood-stage infection recognize different epitopes compared with immune individuals.. Malar J.

[58] Murhandarwati EE, Black CG, Wang L, Weisman S, Koning-Ward TF (2008). Acquisition of invasion-inhibitory antibodies specific for the 19-kDa fragment of merozoite surface protein 1 in a transmigrant population requires multiple infections.. J Infect Dis.

[59] Murhandarwati EE, Wang L, Black CG, Nhan DH, Richie TL (2009). Inhibitory antibodies specific for the 19-kilodalton fragment of merozoite surface protein 1 do not correlate with delayed appearance of infection with *Plasmodium falciparum* in semi-immune individuals in Vietnam.. Infect Immun.

[60] Dent AE, Chelimo K, Sumba PO, Spring MD, Crabb BS (2009). Temporal stability of naturally acquired immunity to Merozoite Surface Protein-1 in Kenyan adults.. Malar J.

[61] Corran PH, O'Donnell RA, Todd J, Uthaipibull C, Holder AA (2004). The fine specificity, but not the invasion inhibitory activity, of 19-kilodalton merozoite surface protein 1-specific antibodies is associated with resistance to malarial parasitemia in a cross-sectional survey in The Gambia.. Infect Immun.

[62] Michon P, Cole-Tobian JL, Dabod E, Schoepflin S, Igu J (2007). The risk of malarial infections and disease in Papua New Guinean children.. Am J Trop Med Hyg.

[63] Lin E, Tavul L, Michon P, Richards JS, Dabod E (2010). Minimal association of common red blood cell polymorphisms with *Plasmodium falciparum* infection and uncomplicated malaria in Papua New Guinean school children.. Am J Trop Med Hyg.

[64] Dutta S, Kaushal DC, Ware LA, Puri SK, Kaushal NA (2005). Merozoite surface protein 1 of Plasmodium vivax induces a protective response against Plasmodium cynomolgi challenge in rhesus monkeys.. Infect Immun.

[65] Cooper JA, Cooper LT, Saul AJ (1992). Mapping of the region predominantly recognized by antibodies to the *Plasmodium falciparum* merozoite surface antigen MSA 1.. Mol Biochem Parasitol.

[66] Persson KE, McCallum FJ, Reiling L, Lister NA, Stubbs J (2008). Variation in use of erythrocyte invasion pathways by *Plasmodium falciparum* mediates evasion of human inhibitory antibodies.. J Clin Invest.

[67] Persson KE, Lee CT, Marsh K, Beeson JG (2006). Development and optimization of high-throughput methods to measure *Plasmodium falciparum*-specific growth inhibitory antibodies.. J Clin Microbiol.

[68] McCallum FJ, Persson KE, Mugyenyi CK, Fowkes FJ, Simpson JA (2008). Acquisition of growth-inhibitory antibodies against blood-stage *Plasmodium falciparum*.. PLoS ONE.

[69] Wilson DW, Crabb BS, Beeson JG (2010). Development of fluorescent *Plasmodium falciparum* for *in vitro* growth inhibition assays.. Malar J.

[70] Beeson JG, Brown GV, Molyneux ME, Mhango C, Dzinjalamala F (1999). *Plasmodium falciparum* isolates from infected pregnant women and children are associated with distinct adhesive and antigenic properties.. J Infect Dis.

[71] Riley EM, Allen SJ, Wheeler JG, Blackman MJ, Bennett S (1992). Naturally acquired cellular and humoral immune responses to the major merozoite surface antigen (PfMSP1) of *Plasmodium falciparum* are associated with reduced malaria morbidity.. Parasite Immunol.

[72] al-Yaman F, Genton B, Kramer KJ, Chang SP, Hui GS (1996). Assessment of the role of naturally acquired antibody levels to *Plasmodium falciparum* merozoite surface protein-1 in protecting Papua New Guinean children from malaria morbidity.. Am J Trop Med Hyg.

[73] Egan AF, Morris J, Barnish G, Allen S, Greenwood BM (1996). Clinical immunity to *Plasmodium falciparum* malaria is associated with serum antibodies to the 19-kDa C-terminal fragment of the merozoite surface antigen, PfMSP-1.. J Infect Dis.

[74] Shi YP, Sayed U, Qari SH, Roberts JM, Udhayakumar V (1996). Natural immune response to the C-terminal 19-kilodalton domain of *Plasmodium falciparum* merozoite surface protein 1.. Infect Immun.

[75] Shi YP, Udhayakumar V, Oloo AJ, Nahlen BL, Lal AA (1999). Differential effect and interaction of monocytes, hyperimmune sera, and immunoglobulin G on the growth of asexual stage *Plasmodium falciparum* parasites.. Am J Trop Med Hyg.

[76] Hodder AN, Crewther PE, Anders RF (2001). Specificity of the protective antibody response to apical membrane antigen 1.. Infect Immun.

