# Supplementary material for: Quantifying the Importance of MSP1-19 as a Target of Growth-Inhibitory and Protective Antibodies against Plasmodium falciparum in Humans
Source: PLoS One. 2011 Nov 15;6(11):e27705. doi: 10.1371/journal.pone.0027705 (PMC3217002; doi:10.1371/journal.pone.0027705)
Supplement: Table S1 — Summary of PfMSP1-19 specific inhibition levels reported in different studies. a S = serum. P = plasma. b M = microscopy based assay. F = flow cytometry based assay. H = Hypoxanthine uptake assay. c Microscopy based assays for Bagabag Island and Madang samples were completed using the D10 wildtype line. Hypoxanthine uptake based assays for Bagabag Island and Madang samples were completed using the transfected D10-PfM3′ line [54]. d Interpretation from Figures 2C and 2D and information in text. d Inhibition minimum at 6 months (16% n = 24) maximum at 24–30 months (29%, n = 19). e Inhibition range for 9 of 20 returned travelers considered to have significant PfMSP1-19 specific inhibition. Data for remaining 11 travelers not reported. f Interpretation from Figures 2B and 3. Spaghetti plots of timepoints for all samples available in supplementary file 4 of the manuscript. g Calculated median for all 137 samples listed in table 1 of the manuscript. Calculated medians for the 3 timepoints are t = 0 (9%, n = 65), t = 1 (10%, n = 37), t = 28 (−14%, n = 35). NR = not reported in manuscript. (DOCX) [file pone.0027705.s001.docx]

**Supplementary Table 1.** Summary of PfMSP1-19 specific inhibition levels reported in different studies.

| Samples | Age | Sample**^a^** | Count***^b^*** | Mean/  ***Median*** | Inhibitory Range | Publication |
| --- | --- | --- | --- | --- | --- | --- |
| Bagabag Is  PNG ***^c^*** | Adults | ***S*** | ***M*** | 25.6% | NR | [54] |
| Bagabag Is  PNG ***^c^*** | Adults | ***S*** | ***H*** | 24.8% | NR | [54] |
| Madang  PNG ***^c^*** | Adults | ***S*** | ***M*** | 23.3% | NR | [54] |
| Madang  PNG ***^c^*** | Adults | ***S*** | ***H*** | 41.4% | NR | [54] |
| Kenyan Highlands | Adults and Children | ***P*** | ***M*** | ***31.5%*** | ≈negative 10% to 75%***^d^*** | [55] |
| The Gambia | 1-70 years | ***P*** | ***M*** | ***63.5% (of total inhibition)*** | NR | [61] |
| Kenya | Birth to 30 months | ***P*** | ***M*** | 16% to 29%***^e^*** | NR | [56] |
| Returned Travelers | Adults | ***S*** | ***M*** | NR | 14.8% to 48.7%***^f^*** | [57] |
| Northwestern Papua, Indonesia | 6-58 | ***S*** | ***M*** | 16% (post 2^nd^ infection) | *negative* 97% to 46% | [58] |
| Western Kenya | Adults | ***P*** | ***F*** | NR | *<10% to >2%*  *(mean 6 timepoints, n=16****^g^)*** | [60] |
| Vietnam | 9-55 years | ***S*** | ***F*** | ***5%^h^*** | *Negative 70% to 68%* | [59] |

***^a^*** ***S***= serum. ***P***= plasma.

***^b^*** ***M***= microscopy based assay. ***F***= flow cytometry based assay. ***H***= Hypoxanthine uptake assay.

***^c^*** Microscopy based assays for Bagabag Island and Madang samples were completed using the D10 wildtype line. Hypoxanthine uptake based assays for Bagabag Island and Madang samples were completed using the transfected D10-PfM3’ line [54].

***^d^*** Interpretation from figures 2C and 2D and information in text.

***^d^*** Inhibition minimum at 6 months (16% n=24) maximum at 24-30 months (29%, n=19).

***^e^*** Inhibition range for 9 of 20 returned travelers considered to have significant PfMSP1-19 specific inhibition. Data for remaining 11 travelers not reported.

***^f^*** Interpretation from figures 2B and 3. Spaghetti plots of timepoints for all samples available in supplementary file 4 of the manuscript.

***^g^*** Calculated median for all 137 samples listed in table 1 of the manuscript. Calculated medians for the 3 timepoints are t=0 (9%, n=65), t=1 (10%, n=37), t=28 (-14%, n=35).

NR = not reported in manuscript.
